# Neuroprotective Strategies in Coronary Artery Disease Interventions

**DOI:** 10.3390/jcdd12040143

**Published:** 2025-04-08

**Authors:** Maurish Fatima, Akbar Bazarbaev, Asama Rana, Ruman Khurshid, Victory Effiom, Nyle Khalid Bajwa, Afsheen Nasir, Katherine Candelario, Sair Ahmad Tabraiz, Samantha Colon, Chanseo Lee, Sedem Dankwa, Irbaz Hameed

**Affiliations:** Division of Cardiac Surgery, Department of Surgery, Yale University School of Medicine, New Haven, CT 06510, USA; maurishfatima16@gmail.com (M.F.); bazarbaevakbar99@gmail.com (A.B.); asama.rana@yale.edu (A.R.); rumankhurshid@gmail.com (R.K.); veffiom24@gmail.com (V.E.); nylebinkhalid@gmail.com (N.K.B.); afsheen.nasir@yale.edu (A.N.); katherinemarinac@gmail.com (K.C.); tabraiz.sairahmad@mayo.edu (S.A.T.); sammyscolon@gmail.com (S.C.); chanseo.lee@yale.edu (C.L.); sedem.dankwa@yale.edu (S.D.)

**Keywords:** coronary artery disease, neuroprotection, stroke

## Abstract

Neuroprotective strategies in coronary artery interventions are essential due to the rising number of high-risk patients undergoing procedures like coronary artery bypass grafting (CABG), totally endoscopic coronary artery bypass (TECAB), and hybrid revascularization. In this review article, we summarize the neurological complications associated with coronary artery disease intervention and the risk mitigation strategies. CABG carries significant risks, including ischemic stroke, encephalopathy, seizures, and peripheral nerve injuries. Risk factors include advanced age, hypertension, diabetes, and atherosclerosis. Off-pump CABG minimizes stroke risk by avoiding aortic manipulation and CPB. TECAB and hybrid revascularization have fewer reported neurological complications but still pose risks of stroke and cranial nerve injuries. Pharmacological neuroprotection includes agents such as barbiturates, volatile anesthetics, lidocaine, NMDA receptor antagonists, magnesium, nimodipine, corticosteroids, and aprotinin. Deep hypothermic circulatory arrest (DHCA) is reserved for complex aortic cases requiring a bloodless surgical field. Intraoperative strategies involve cerebral perfusion monitoring, embolic protection devices, and therapeutic hypothermia. Preoperative optimization targets risk factors, arrhythmia prevention, and antiplatelet therapy management. Postoperatively, timely antiplatelet administration, glucose control, hemodynamic stabilization, and cognitive monitoring are critical. Comprehensive neuroprotective approaches, spanning pre- to postoperative phases, aim to reduce neurological complications and enhance outcomes in coronary interventions.

## 1. Mechanism of Cardiac Surgery Related Brain Damage

An increase in the volume of cardiac surgery has led to a greater proportion of high-risk patients undergoing coronary artery interventions, underscoring the need for neuroprotective strategies to mitigate perioperative and postoperative neurological complications, particularly in cardiothoracic procedures.

**Figure 1:**
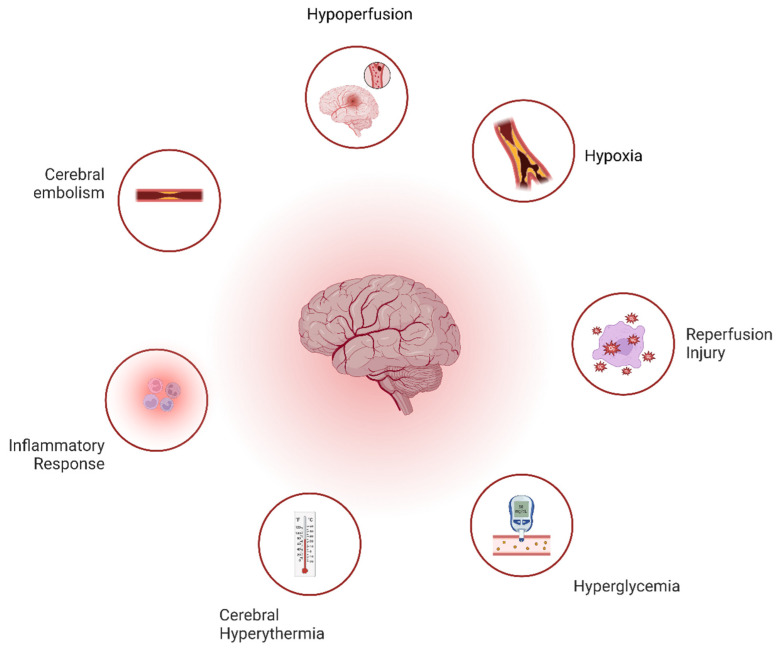
Pathophysiology of the neurological deficit during cardiac surgery.

The mechanism of cardiac surgery-related brain damage is multifactorial, primarily involving ischemic causes like cerebral hypo-perfusion and embolism (Figure 1). Key mechanisms include the following:

1. Altered Cerebral Perfusion: Cerebral hypoperfusion during cardiopulmonary bypass (CPB) leads to ischemic injury, exacerbated by a reduced clearance of micro-emboli. Reperfusion generates reactive oxygen species (ROS), causing neuronal death [1].
2. Hypoxia-Related Injury: Hypoperfusion-induced hypoxia triggers molecular pathways (e.g., HIF activation), ATP depletion, ion pump failure, and cell swelling, culminating in neuronal injury [1].
3. Reperfusion Injury: ROS production during reperfusion damages neurons via protein nitrosylation and mitochondrial dysfunction [1,2].
4. Cerebral Embolism: Macro- and micro-emboli, arising from atherosclerotic plaques, gaseous emboli, or surgical debris obstruct cerebral blood flow, contributing to cognitive dysfunction [1].
5. Inflammatory Response: CPB triggers systemic inflammation, causing blood–brain barrier leakage, cerebral edema, and neuronal damage.
6. Cerebral Hyperthermia: Brain overheating during CPB exacerbates neuronal death, particularly in ischemic regions [2].
7. Hyperglycemia: Stress-induced hyperglycemia increases ROS production, promotes inflammation, and causes metabolic acidosis, contributing to neuronal injury [1,2].

Each mechanism underscores the complexity of brain injury associated with cardiac surgery and highlights potential targets for intervention.

## 2. Neurological Risk Associated with Different Types of Coronary Artery Surgery

### 2.1. Neurological Complications in Coronary Artery Bypass Grafting (CABG)

#### 2.1.1. Stroke

Stroke remains a significant concern in CABG, especially when cardiopulmonary bypass (CPB) is used. Aortic manipulation can dislodge atheroemboli, potentially causing ischemic stroke [1]. Patients with extensive atherosclerosis or calcified aortas are at increased risk, necessitating careful preoperative assessment and surgical planning [2]. The reported incidence of ischemic stroke during CABG ranges from 2% to 6% [3,4]. In earlier CABG studies, 40% of strokes occurred intraoperatively, with postoperative strokes peaking around 40 h [5]. Sources of emboli include cardiac arrhythmias such as atrial fibrillation, septal aneurysms, and regions of reduced left ventricular function.

Several predictors of postoperative stroke have been identified, including hypertension, diabetes, and prior stroke [6,7]. Advancing age is among the strongest predictors: compared with patients under 65, the risk is 4.6-fold higher for those aged 65–75 and 5.2-fold higher for those over 75 [8]. Female patients also appear to have a higher risk of postoperative cerebral ischemia and mortality after CABG [9,10]. Genetic predispositions may influence stroke risk, as polymorphisms in inflammatory markers (e.g., C-reactive protein, interleukin-6) have been associated with a threefold increase in postoperative stroke [11]. While an Apolipoprotein E ε4 allele has been linked to postoperative cognitive dysfunction in some studies, other investigations have not confirmed this relationship [12,13].

#### 2.1.2. Encephalopathy

Encephalopathy after cardiac surgery often has a multifactorial etiology similar to that of stroke and may be influenced by medications (e.g., benzodiazepines) and metabolic disturbances. Originally described as confusion and altered consciousness, the term now encompasses a broad spectrum from delayed emergence from anesthesia to prolonged stupor [3,14,15]. The incidence ranges from 18% to 28%, but it is often transient [3,16].

#### 2.1.3. Seizures

Seizures are relatively rare but can occur postoperatively due to factors such as acute stroke, medication effects, contrast-induced encephalopathy, or hypoxic brain injury. In one study, postoperative seizures occurred in 0.95% of CABG patients, often within the first few hours after surgery, and correlated with prolonged ICU stays and higher ICU mortality [17]. Less than 0.5% of patients on CPB develop postoperative seizures [18]. Procedures involving aortic arch manipulation and periods of deep hypothermic circulatory arrest are at higher risk [19]. Other factors include drugs that lower seizure threshold, alcohol or benzodiazepine withdrawal, metabolic derangements, and the use of high-dose tranexamic acid [20,21].

#### 2.1.4. Peripheral Nerve Injury

Peripheral nerve dysfunction due to operative positioning or retraction is more common in CABG than in many other procedures. About 13% of patients experience new peripheral nerve injuries, most frequently brachial plexopathies related to internal mammary artery harvesting [22,23]. Saphenous nerve injuries can also occur during vein harvesting, which results in numbness and discomfort in the distribution of this superficial sensory nerve [24].

### 2.2. Neurological Complications in Totally Endoscopic Coronary Artery Bypass (TECAB)

Neurological complications and outcomes in TECAB are less frequently reported. A retrospective analysis of 1500 TECAB patients noted neurological symptoms in 5.53%, including cerebrovascular accidents (1.27%) and transient ischemic attacks (0.53%), as well as seizures and delirium [24]. Another study reported a stroke incidence of approximately 1.7% after TECAB [25]. Early multicenter data suggested freedom from major adverse cardiac and cerebral events at 6 months ranging from 91.2% to 94.9%, and about 75.6% at 5 years in selected cohorts [26,27].

### 2.3. Neurological Complications in Hybrid Coronary Revascularization (HCR)

Hybrid coronary revascularization (HCR) combines surgical and percutaneous techniques like minimally invasive direct CAB (MIDCAB) and PCI of the non-left anterior descending artery (LAD) to address multi-vessel coronary artery disease. Studies have shown an that HCR is associated with an increased incidence of cerebral infarction and cranial nerve injury. Understanding these risks is crucial for optimizing patient outcomes. HCR procedures can lead to cranial nerve injury due to surgical manipulation in thoracic approaches. Patients receiving hybrid operations have an increased risk of cerebral infarction, particularly those with higher SYNTAX scores thus indicating more complicated coronary disease. And long term outcomes have shown a composite endpoint of major adverse cardiac and cerebrovascular events (MACCE). However, HCR has been found to be associated with a lower mortality rate and improved graft patency, suggesting that careful patient selection and risk stratification can mitigate potential complications [28,29,30].

Neuroprotective strategies in the coronary artery disease interventions have been summarized in Table 1.

## 3. Pharmacological Brain Protection Strategies

### 3.1. Barbiturates

Barbiturates reduce cerebral metabolic oxygen demand (CMRO2), enhance ischemic tolerance, and improve cerebrovascular parameters during CPB. Their use in combination with hypothermia can significantly reduce the risk of brain ischemia in cardiac surgery. T. Hirotani’s study showed that the use of thiopental paired with hypothermia during cardiac surgery can significantly reduce the risks of brain ischemia [31,32,33,34].

### 3.2. Volatile Anesthetics

Volatile anesthetics (isoflurane, sevoflurane) act through activation of TWIK-related acid-sensitive potassium channel (TASK) channels in cortical neurons, causing membrane hyperpolarization. They activate the PI3-Akt pathway, interact with N-methyl-D-aspartate receptors (NMDARs) receptors, and have anti-inflammatory effects. They are effective even with post-ischemic application, especially at a concentration of 1 MAC [35].

### 3.3. Lidocaine

Lidocaine protects through slowing ischemic transmembrane ion shift, reducing cerebral metabolism, and decreasing excitotoxin release. Regarding the timing of lidocaine administration, there are no specific conclusions. There are studies that differ in the timing of lidocaine administration during cardiac surgery: two studies over 48 h showed good results in brain protection in non-diabetic patients, but not in diabetics. In one study, lidocaine showed no protective effect with 12 h administration, but another study showed positive results when used until the end of surgery [33,35,36,37].

### 3.4. N-Methyl-D-aspartate (NMDA) Receptor Antagonist

NMDA antagonists block excite-toxic damage, regulate apoptotic proteins, and suppress inflammation [38]. A study of patients after cardiac surgery showed that a single administration of ketamine during anesthesia induction significantly reduces the risk of postoperative cognitive disorders [39]. Remacemide also showed significant neuroprotection in cardiac surgical interventions. Xenon improved neurological function in mice, but in patients undergoing cardiac surgery, it did not lead to a significant reduction in postoperative delirium [40,41,42].

### 3.5. Magnesium

Magnesium regulates vascular tone and circulation. Low magnesium levels increase the risk of ischemia. In cardiac surgery, magnesium can protect the brain from ischemia especially in the early stage, and also improve functional neurological outcomes [43,44,45,46].

### 3.6. Nimodipine

Nimodipine dilates cerebral vessels, crosses the blood–brain barrier. In Forsman’s study, patients after cardiac surgery with cardiopulmonary bypass received nimodipine or placebo. The nimodipine group showed better results in speech and memory tests [47,48,49,50].

### 3.7. Corticosteroids

Corticosteroids suppress inflammation. Preliminary systemic administration of steroids significantly reduces total body edema and cerebral vascular permeability, shows better immune-histochemical indicators of neuroprotection after deep hypothermic circulatory arrest during cardiac surgery. Lower levels of neuron-specific enolase in patients receiving methylprednisolone suggest the benefit of corticosteroids for reducing neuronal damage during cardiac surgery [51,52,53].

### 3.8. Aprotinin

Aprotinin is used in cardiac surgery to reduce blood loss and preserve platelet function. It reduces inflammation and improves neurological outcomes after prolonged periods of deep hypothermic circulatory arrest or low-flow cardiopulmonary bypass [54].

### 3.9. Deep Hypothermic Circulatory Arrest (DHCA)

DHCA substantially lowers cerebral metabolic demand—at 18 °C, cerebral metabolism reaches only 12–25% of normal [55]. It suppresses glutamate release and reduces ATP breakdown and lactate accumulation. However, the role of DHCA in routine coronary artery surgery is limited. DHCA is not typically required for standard CABG or PCI, as these procedures do not routinely involve operations on the ascending aorta or arch that mandate a bloodless field for reconstruction.

DHCA is most applicable in complex aortic arch procedures, including cases of extensive atherosclerosis of the ascending aorta or when repairing aneurysms or dissections involving the arch vessels. In such scenarios, DHCA provides a motionless, bloodless field to facilitate precise surgical work while minimizing cerebral injury. Its use in coronary revascularization is therefore confined to exceptional circumstances, such as combined procedures involving the arch or severe atheromatous disease where alternative neuroprotective methods are insufficient [55,56].

## 4. Intra-Operative Neurological Risk Management in TECAB, and CABG

Intra-operative management during coronary procedures such as, TECAB, and CABG presents distinct neurologic injury risks. These risks primarily stem from embolic events, ischemia–reperfusion injury, and hemodynamic instability, which can lead to complications like stroke, cognitive dysfunction, or transient ischemic attacks Neurological outcomes are closely tied to the surgical techniques employed, the patient’s pre-existing conditions, and the intra-operative management strategies used to mitigate these risks [2,57]. Advanced cerebral monitoring and strategic planning can reduce these risks.

### 4.1. Risk Mitigation Strategies

#### 4.1.1. Cerebral Perfusion Monitoring

One of the most important aspects of intra-operative management in both CABG and TECAB is the maintenance of cerebral perfusion. Techniques such as near-infrared spectroscopy (NIRS) or transcranial Doppler ultrasonography can be used to monitor cerebral oxygenation and blood flow during surgery [58]. In CABG, particularly when CPB is used, maintaining adequate perfusion pressures and minimizing periods of low-flow states are essential to reducing the risk of ischemic injury [59].

#### 4.1.2. Embolic Protection Strategies

For patients undergoing CABG, especially with atherosclerotic or calcified aortas, “no-touch” aortic strategies—where the aorta is not manipulated—can reduce the incidence of embolic stroke [18,60]. Epiaortic ultrasound imaging can identify aortic plaques, guiding cannulation and graft anastomosis locations to prevent emboli. By avoiding heavily diseased aortic segments, surgeons can reduce stroke risk.

#### 4.1.3. Off-Pump CABG (OPCAB)

Off-pump CABG avoids CPB and reduces embolic load and inflammatory response, potentially lowering neurological complications. Without the need for an aortic cross-clamp, surgeons can minimize plaque disruption and preserve cerebral autoregulation, leading to improved neurocognitive outcomes compared to traditional on-pump CABG [61].

#### 4.1.4. Neuroprotective Anesthesia and Pharmacology

The intra-operative use of anesthetics with neuroprotective properties, such as dexmedetomidine, has been suggested to minimize neuronal injury by reducing inflammation and metabolic demand during surgery. Both CABG and TECAB benefit from anesthetic agents that maintain stable hemodynamics and reduce the stress response, which in turn decreases the likelihood of cerebral ischemia. The use of statins and antiplatelet agents before and after surgery can further decrease embolic stroke risks by stabilizing atherosclerotic plaques and reducing clot formation [62,63].

#### 4.1.5. Therapeutic Hypothermia

For patients undergoing high-risk procedures, especially those experiencing cardiac arrest during PCI, therapeutic hypothermia is a neuroprotective strategy that lowers metabolic demand, reduces inflammation, and protects neuronal tissue. This strategy has been shown to improve neurological outcomes in cardiac surgery patients, particularly those with compromised brain perfusion [59,63]. While deep hypothermia is less common in routine coronary revascularization, mild hypothermia is sometimes used for neuroprotection during complex procedures.

## 5. Pre- and Postoperative Neuroprotective Strategies in CABG

Most recent studies emphasize both pharmacological and procedural interventions across pre-operative, intra-operative, and post-operative stages to prevent neurological complications such as stroke in cardiac surgery. Managing risk factors and comorbidities also play a major role in preventing these complications.

### 5.1. Preoperative Strategies

According to 2024 The European Association for Cardio-Thoracic Surgery (EACTS_ guidelines on Perioperative Medication in Adult Cardiac Surgery, patients already taking statins should continue statin therapy at preoperative dose (class IIA recommendation). However, it is not recommended to initiate statin therapy shortly before elective cardiac surgery due to the associated risk of acute renal failure (class III recommendation).

For post-CABG patients not achieving low-density lipoprotein–cholesterol (LDL-C) levels <55 mg/dL, despite high-dose statins, adding ezetimibe is recommended [64]. For patients not achieving LDL-C <55 mg/dL on high-dose statins, adding ezetimibe is recommended (IMPROVE-IT trial) [65].

According to 2024 EACTS guidelines on Perioperative Medication in Adult Cardiac Surgery, patients undergoing CABG who are on low-dose ASA preoperatively, continuing ASA throughout the perioperative period is recommended to reduce ischemic events (Class I recommendation).

In CABG patients who are ASA-naive or have discontinued ASA, preoperative initiation shows no clear benefit. The ATACAS (Aspirin and Tranexamic Acid for Coronary Artery Surgery) trial found no difference in death or thrombotic complications at 30 days, nor in bleeding risk, between those given 100 mg ASA preoperatively and those on a placebo [64,66].

Prophylaxis against perioperative arrhythmias is paramount, as atrial fibrillation (AF) is associated with increased stroke risk. Beta-blockers (BBs) and amiodarone are recommended to reduce postoperative AF incidence, though amiodarone use must be balanced against its long-term complications. Initiating beta-blockers (BBs) before surgery to prevent arrhythmias requires caution, since conclusive evidence supporting the benefits of initiating BBs shortly before the operation is still lacking. If BBs are to be administered preoperatively in naive patients, a gradual adjustment of the dose is recommended (class IIB recommendation), using short-acting drugs and formulations based on the patient’s blood pressure and heart rate and starting several days before the operation. Although magnesium, fish oil and omega-3 fatty acids are thought to prevent POAF, RCTs provide conflicting evidence, preventing a definitive recommendation [67,68].

### 5.2. Intraoperative Techniques

As discussed, employing off-pump techniques, epiaortic ultrasound, embolic protection devices, careful anesthetic management, and maintaining stable hemodynamics all contribute significantly to neuroprotection.

### 5.3. Postoperative Management

According to 2024 EACTS guidelines on Perioperative Medication in Adult Cardiac Surgery, in patients undergoing CABG, it is recommended to (re)start low-dose ASA within 24 h (ideally < 6 h) postoperatively to reduce ischemic events and graft occlusion (Class I Recommendation). The early initiation of low-dose ASA after CABG is associated with a reduced risk of death and ischemic complications and should be continued indefinitely in patients who do not have contraindications to ASA. Current guidelines recommend DAPT, which has been associated with reduced risk of graft failure, reduced all-cause mortality and ischemic events after CABG in patients with ACS [61].

Targeted therapy to prevent postoperative arrhythmias, along with strict blood pressure and glycemic control, can further reduce cerebral risk. Measures to prevent delirium—adequate pain control, appropriate sedation management, environmental optimization—help improve long-term neurological function.

### 5.4. Long-Term Follow-Up

Long-term follow-up with imaging, neurocognitive testing, and continued risk-factor modification (lipid management, blood pressure control, smoking cessation, diabetes management) is essential. These strategies ensure sustained neuroprotection and improved quality of life.

## 6. Conclusions

Neurological complications remain a critical consideration in coronary artery interventions, including CABG, TECAB, and HCR. Their mitigation requires a multidimensional approach: careful patient selection, meticulous surgical technique, advanced cerebral monitoring, judicious use of pharmacological adjuncts, and robust perioperative care. Techniques such as epiaortic ultrasound, off-pump CABG, and strict embolic protection strategies, along with pre- and postoperative optimization of risk factors and therapies, improve neurological outcomes. As evidence evolves, these neuroprotective strategies continue to refine and enhance long-term patient well-being.

## Figures and Tables

**Table 1:** Summary of neuroprotective strategies in coronary artery interventions.

Strategy/Factor	Application in Coronary Interventions	Rationale/Mechanism	Key Considerations
Surgical Approach	CABG, TECAB, HCR, PCI	Minimizing aortic manipulation (e.g., “no-touch” technique) reduces risk of embolization and stroke	Patient selection, preoperative imaging (e.g., epiaortic ultrasound), and careful graft planning
Off-Pump CABG (OPCAB)	CABG	Avoids cardiopulmonary bypass, reducing embolic load and inflammatory response	Suitable for patients at high neurological risk; surgeon expertise required
Epiaortic Ultrasound	CABG	Identifies aortic plaque burden and guides safe cannulation/anastomosis sites	Reduces stroke risk by preventing plaque dislodgment
Deep Hypothermic Circulatory Arrest (DHCA)	Complex aortic/arch procedures rarely involving coronary surgery	Lowers cerebral metabolism and protects the brain in scenarios with no blood flow (e.g., arch aneurysm repair)	Not routine for standard coronary procedures; reserved for complex aortic reconstructions
Pharmacologic Agents	CABG, PCI, TECAB, HCR	Reduce inflammation, excitotoxicity, and metabolic demand	Agents include volatile anesthetics, barbiturates, lidocaine, NMDA antagonists, magnesium, nimodipine, corticosteroids, aprotinin
Anesthetic Management	All coronary interventions	Neuroprotective anesthetics (e.g., volatile agents, dexmedetomidine) stabilize hemodynamics and reduce cerebral metabolic rate	Tailor anesthetic choice to patient comorbidities and risk profile
Therapeutic Hypothermia	High-risk CABG/PCI scenarios	Decreases cerebral metabolic demand, limits ischemic injury	Typically mild/moderate hypothermia; must balance benefits against potential coagulopathy and arrhythmias
Cerebral Perfusion Monitoring	CABG, TECAB	NIRS, transcranial Doppler to ensure adequate cerebral blood flow and oxygenation	Early detection of ischemia allows prompt intervention (adjusting perfusion, BP, etc.)
Preoperative Medical Optimization	CABG, PCI	Controlling hypertension, diabetes, optimizing lipids (statins, ezetimibe) reduces stroke risk	Follow guidelines for continuation of statins, careful initiation of new therapies pre-surgery
Antiplatelet Management	CABG, PCI	ASA and/or P2Y12 inhibitors reduce thrombotic events; timing is critical to balance bleeding and ischemic risks	Tailored per guidelines (e.g., DAPT bridging, test platelet function if recently discontinued)
Arrhythmia Prophylaxis	CABG, PCI	Beta-blockers, amiodarone reduce incidence of postoperative AF and associated stroke risk	Start/continue BBs if already on therapy; carefully initiate in naïve patients
Postoperative Therapies	CABG, PCI	Early ASA (within 6 h), resuming DAPT in ACS/PCI patients reduce graft occlusion and ischemic events	Monitor bleeding risk, ensure stable hemodynamics, and address delirium prevention
Long-Term Follow-Up	All interventions	Ongoing risk factor management, neurocognitive assessments, and lipid control support long-term neuroprotection	Regular follow-up imaging, medications adherence, lifestyle interventions

## Data Availability

No new data were created or analyzed in this study. Data sharing is not applicable.

## Abbreviations

The following abbreviations are used in this manuscript:

CABG	Coronary artery bypass grafting
TECAB	Totally endoscopic coronary artery bypass
CPB	Cardiopulmonary bypass
HCR	Hybrid coronary revasculariztion
CMRO2	Cerebral metabolic oxygen demand
DHCA	Deep hypothermic circulatory arrest
OPCAB	Off-pump CABG
